# COVID-19 in Egypt after a year: the first and second pandemic waves from the radiological point of view; multi-center comparative study on 2000 patients

**DOI:** 10.1186/s43055-021-00549-3

**Published:** 2021-07-08

**Authors:** Ahmed Samir, Amr Magdy Elabd, Walid Mohamed, Ayman Ibrahim Baess, Rania Ahmed Sweed, Mohamed Saied Abdelgawad

**Affiliations:** 1 Department of Radio-diagnosis, Faculty of Medicine, Alexandria University, Alexandria, Egypt; 2 Department of Radio-diagnosis, Medical Research Institute, Alexandria University, Alexandria, Egypt; 3 Department of Chest diseases, Faculty of Medicine, Alexandria University, Alexandria, Egypt; 4 Department of Radio-diagnosis and Intervention, National Liver Institute, University of Menoufia, Shibin Al Kawm, Egypt

**Keywords:** COVID-19, Egypt, MSCT, Pandemic, Waves

## Abstract

**Background:**

One year has passed since the announcement of COVID-19 as a pandemic and two waves had already stricken Egypt. The authors witnessed several atypical radiological features through the second pandemic wave, either early at the active infective stage or delayed at the post-infectious convalescent period. They believed every radiologist should be familiar with these features. Therefore, they performed this comparative study on 2000 Egyptian patients using multi-slice computed tomography (MSCT) to highlight the radiological differences between the first and second pandemic waves and correlate them to the clinical status.

**Results:**

This random multi-center comparative study was retrospectively conducted on 2000 COVID-19 Egyptian patients; 1000 patients were registered at the first pandemic wave from April 2020 till September 2020, while the other 1000 patients were registered at the second pandemic wave from October 2020 till March 2020. Follow up CT examinations were performed for 49 and 122 patients through the first and second pandemic waves respectively. MSCT examinations were carefully evaluated by four expert consulting radiologists who came to a consensus. Meanwhile, the correlation with the clinical outcome was performed by two consulting pulmonologists. During the second pandemic wave, the prevalence rate of the “crazy-paving” pattern had significantly increased by 1.3 times (*P* value = 0.002). Additionally, the prevalence rate of the “air-bubble” sign had significantly increased by 1.9 times (*P* value = 0.02). Similarly, the presence of enlarged mediastinal lymph nodes (> 1 cm in short-axis diameter) had significantly increased by 1.7 times (*P* value = 0.036). Furthermore, the prevalence rate of pericardial effusion had significantly increased by 2.5 times (*P* value = 0.003). The above-mentioned signs were correlated to increased clinical severity and higher rates of hospitalization. Unexpectedly, other atypical radiological signs were only encountered through the second pandemic wave, including bronchiectatic changes (2.5%), “head-cheese” pattern (0.8%), cavitation (0.5%), and “bulls-eye” sign (0.2%). The prevalence rate of post-COVID fibrosis had doubled through the second wave but not in a significant way (*P* value = 0.234). Secondary fungal infection was only encountered throughout the second pandemic wave in four patients. COVID-19 reinfection was encountered in a single patient only during the second pandemic wave.

**Conclusion:**

After 1 year from the announcement of COVID-19 as a pandemic, the radiological presentation of COVID-19 patients showed some significant differences between its first and second waves.

## Background

Around 1 year has passed since Egypt had reported the first case of COVID-19 infection in Africa on February 14, 2020 [1] and since WHO declared it as a pandemic in March 2020 [2]. On April 21, 2020, 3333 cases of COVID-19 were reported by the Egyptian Ministry of Health [3]. On May 31, 2020, 24,985 cases were furtherly reported [2]. On June 13, 2020, 41,303 cases and 1422 deaths were reported nearly at the peak of the first pandemic wave [4]. On September 2, 2020, 99,280 cases and over 5400 deaths were reported [5] and in mid-October, 2020, the second pandemic wave was announced [6] and reached its peak in December 2020 and January 2021.

Over the past year, the clinical, laboratory, and radiological characteristics of COVID-19 have been thoroughly evaluated by multiple observational studies, systematic reviews, and meta-analysis [7–9]. CT was known as a rapid and sensitive tool for diagnosis of COVID-19 infection but with low specificity [10, 11]. Several typical, indeterminate, and atypical CT-signs of COVID-19 were described [12, 13].

The authors witnessed several atypical radiological features through the second pandemic wave, either early at the active infective stage or delayed at the post-infectious convalescent period. They believed every radiologist should be familiar with these features. Therefore, they performed this comparative study on 2000 Egyptian patients using multi-slice computed tomography (MSCT) to highlight the radiological differences between the first and second pandemic waves and to report their impact on the clinical status.

## Methods

### Study protocol

This random multi-center comparative study was retrospectively conducted on 2000 COVID-19 Egyptian patients; 1000 patients were registered at the first pandemic wave from April 2020 till September 2020, while the other 1000 patients were registered at the second pandemic wave from October 2020 till March 2020. MSCT examinations were carefully evaluated by four expert consulting radiologists who came to a consensus. They have long time experience in chest imaging (15–25 years) and they were informed with all data. Meanwhile, the correlation with the clinical outcome regarding the rate of hospitalization and impact on respiratory functions later was performed by two consulting pulmonologists, who have long time experience in the management of chest diseases (16 and 20 years).

The Ethics Committee of our University hospital had approved the study protocol. The patient consent was waived by the Research Ethics Board with the assurance of the respect of the confidentiality of patients and medical records.

*Inclusion criteria were* as follows: (1) positive PCR and MSCT results for COVID-19, and (2) available clinical records. *Exclusion criteria were as follows:* (1) poor CT images with motion artefacts, and (2) absent clinical records.

### CT machines and scanning parameters

Multiple multi-detector CT machines had performed the MSCT examinations including Philips Brilliant-16 (USA), Siemens SOMATOM Emotion 16 and Siemens SOMATOM Sensation 64 (Germany), Canon Medical Systems; Toshiba Aquilion 64 and Toshiba Aquilion CXL/CX 128 (USA).

The CT Scanning parameters were as follows: slice thickness = 1–2 mm, FOV = 350 mm × 350 mm, tube rotation = 0.6–0.9 second and detector collimation = 1 mm. The irradiation dose parameters were as follows: 120–130 kVp and 100–200 mA (according to the machine type as well as patient age and weight). Intravenous contrast administration was not utilized.

### CT evaluation

Multi-planar reconstruction (MPR) was performed for CT images. Additionally, the maximum intensity projection (MIP) reconstruction was utilized for nodular and reticular assessment, while the minimum intensity projection (Min-IP) reconstruction was utilized for airway assessment and mosaic attenuation characterization.

The following CT features were compared between the patients throughout the first and second pandemic waves:

A. Preexisting lung comorbidity; including COPD, ILDs, lung neoplasms (whether lung cancer or metastatic disease), and miscellaneous conditions (such as TB and sarcoidosis).
B. MSCT findings during the first two weeks of infection, including:

- Ground-glass opacities with or without consolidative changes [14].
- “Atoll sign”: Central ground-glass attenuation with peripheral consolidation [14].
- “Bulls-eye sign” or “Target sign” or “Double-halo”: Central nodule or consolidation and peripheral organization rim with ground-glass in between [15].
- “Crazy-paving pattern”: Ground-glass attenuation with septal thickening [14].
- “Air-bubble sign”: Small foci of air trapping or bronchiolectasis sequel to septal thickening and fibrosis [16, 17].
- Cavitary changes.
- Airway involvement: bronchial wall thickening, bronchiectasis and tree in bud nodules [14].
- “Head-cheese pattern”: ground-glass attenuation with alternating areas of air trapping [14].
- Peri-lobular fibrosis.
- Significant mediastinal or hilar lymph node enlargement (Short axis diameter > 1 cm) [14].
- Pleural effusion.
- Pneumo-mediastinum: spontaneous if not preceded by oxygen therapy.
- Pericardial effusion.

C. CT Severity score: automated quantification of the diseased lung volume was calculated using OsiriX MD 11.0 software (Pixmeo SARL, Geneva, Switzerland). Score 1: referred to 0-25%, score 2: referred to 26–50%, score 3: referred to 51–75%, score 4: > 75% [13].
D. Post-infectious sequel or complications (after 1 month of infection): including residual ground-glass opacities, residual air trapping, or mixed head-cheese pattern [14] as well as post-COVID fibrosis.
E. Signs of secondary bacterial or fungal infection.
F. COVID-19 reinfection.

### Statistical methods

The prevalence rate was estimated. Data were compared using online calculators (https://www.socscistatistics.com) with chi-square test and *p* value analysis (*p* value < 0.05 was considered as statistically significant).

## Results

### Demographic criteria (Table 1)

**Fig. 1:**
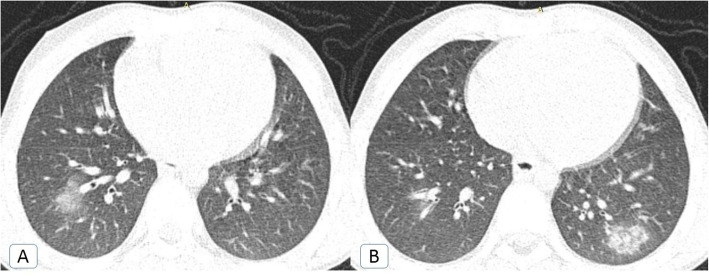
Typical COVID-19 CT signs in a 7-year-old boy: **A**, **B** Axial chest CT cuts (lung window) showing bilateral lower lobar ground glass patches with evolving left basal consolidative changes

Throughout the first and second pandemic waves, the rate of infection was highest in the fourth decade of life. Despite that, the rate of infection through the fourth and fifth decades of life witnessed more than 50% reduction through the second pandemic wave (significant *P* value < 0.00001, for both). On the other hand, the rate of infection through the second and third decades of life during the second wave witnessed around 10 and 1.5 times elevation respectively (*P* value ≤ 0.00001 and 0.001). Additionally, a single 7-year-old patient was also registered (Fig. 1).

**Table 1:** Distribution of COVID-19 patients according to the demographic and radiological features in comparison between the first and second pandemic waves with statistical significance

The demographic and radiological features:	First pandemic wave		Second pandemic wave		*P* value
	*N*	%	*N*	%	
Age:					
• 0–10 years:	–	–	1	0.1%	0.317
• 11–20 years:	15	1.5%	149	14.9%	< *0.00001*
• 21–30 years:	83	8.3%	128	12.8%	*0.001*
• 31–40 years:	333	33.3%	187	18.7%	< *0*.*00001*
• 41–50 years:	292	29.2%	177	17.7%	< *0*.*00001*
• 51–60 years:	125	12.5%	163	16.3%	*0.016*
• 61–70 years:	107	10.7%	132	13.2%	0.085
• 71–80 years:	55	5.5%	63	6.3%	0.448
- Mean:	43		34		
- Standard deviation (SD):	19.6		17.9		
Sex (Male : Female):	62.5 : 37.5%		61 : 39 %		0.771
Pre-existing lung co-morbidity:	13	1.3%	42	4.2%	*0*.*000073*
• COPD:	7	0.7%	22	2.2%	*0*.*005*
• ILD:	–	–	5	0.5%	*0.025*
• TB:	–	–	1	0.1%	0.317
• Sarcoidosis:	4	0.4%	9	0.9%	0.164
• Lung cancer or metastatic process:	2	0.2%	5	0.5%	0.256
CT findings (at two weeks of infection):					
• Ground-glass opacities with or without consolidations:	1000	100%	1000	100%	–
• Atoll sign:	84	8.4%	99	9.9%	0.245
• Bulls-eye or target sign:	–	–	2	0.2%	0.157
• Crazy-paving:	167	16.7%	222	22.2%	*0*.*002*
° *Patchy* lesions with inter-lobular septal thickening.	105	10.5%	142	14.2%	*0*.*012*
° *Diffuse* ground-glass with inter-lobular septal thickening.	62	6.2%	80	8%	0.117
Peri-lobular fibrosis	123	12.3%	173	17.3%	*0*.*02*
• Air-bubble or vacuolar sign:	19	1.9%	36	3.6%	*0*.*02*
• Cavitation (> 1 cm):	–	–	5	0.5%	*0*.*025*
• Air-way involvement:	–	–	33	3.3%	< *0*.*00001*
° Head-cheese:	–	–	8	0.8%	*0*.*005*
° Bronchiectasis:	–	–	25	2.5%	< *0*.*00001*
° Tree in bud nodules	1	0.1%	1	0.1%	1
• Lymph nodes (short axis > 1 cm):	22	2.2%	38	38%	*0*.*036*
• Pleural effusion:	5	0.5%	3	0.3%	0.479
• Pericardial effusion:	13	1.3%	33	3.3%	*0.003*
• Pneumo-mediastinum:	2	0.2%	7	0.7%	0.095
Post-infectious sequel or complications (after 1 month of infection):					
• Residual ground-glass attenuation:	7/49	14.3%	41/122	33.6%	*0.011*
• Fibrosis:	3/49	6.1%	15/122	12.3%	0.234
• Residual head cheese:	–	–	5/122	4.1%	0.151
• Secondary air trapping only:	–	–	3/122	2.5%	0.268
Secondary fungal infection:	–	–	4	0.4%	*0.045*
COVID-19 reinfection:	–	–	1	0.1%	0.317

* *P* value < 0.05 is considered statistically significant

Meanwhile, minimal and insignificant changes were encountered regarding the rate of infection through the sixth and seventh decades of life. Similarly, minimal and insignificant changes were documented regarding sex predilection. Males were more commonly infected during the first and second pandemic waves (62.5% and 61%, respectively).

Higher CT severity scores (> 50%) were depicted mainly in the 6^th^ and 7^th^ decades of life throughout both pandemic waves (93% and 83%, respectively). This coincided with corresponding higher clinical severity and increased rates of hospitalization. During the second pandemic wave, around 10% rise in the clinical and CT severity-scores as well as the hospitalization rates were reported through the 4^th^ and 5^th^ decades of life.

### Preexisting lung comorbidity and clinical severity (Table 1)

**Fig. 2:**
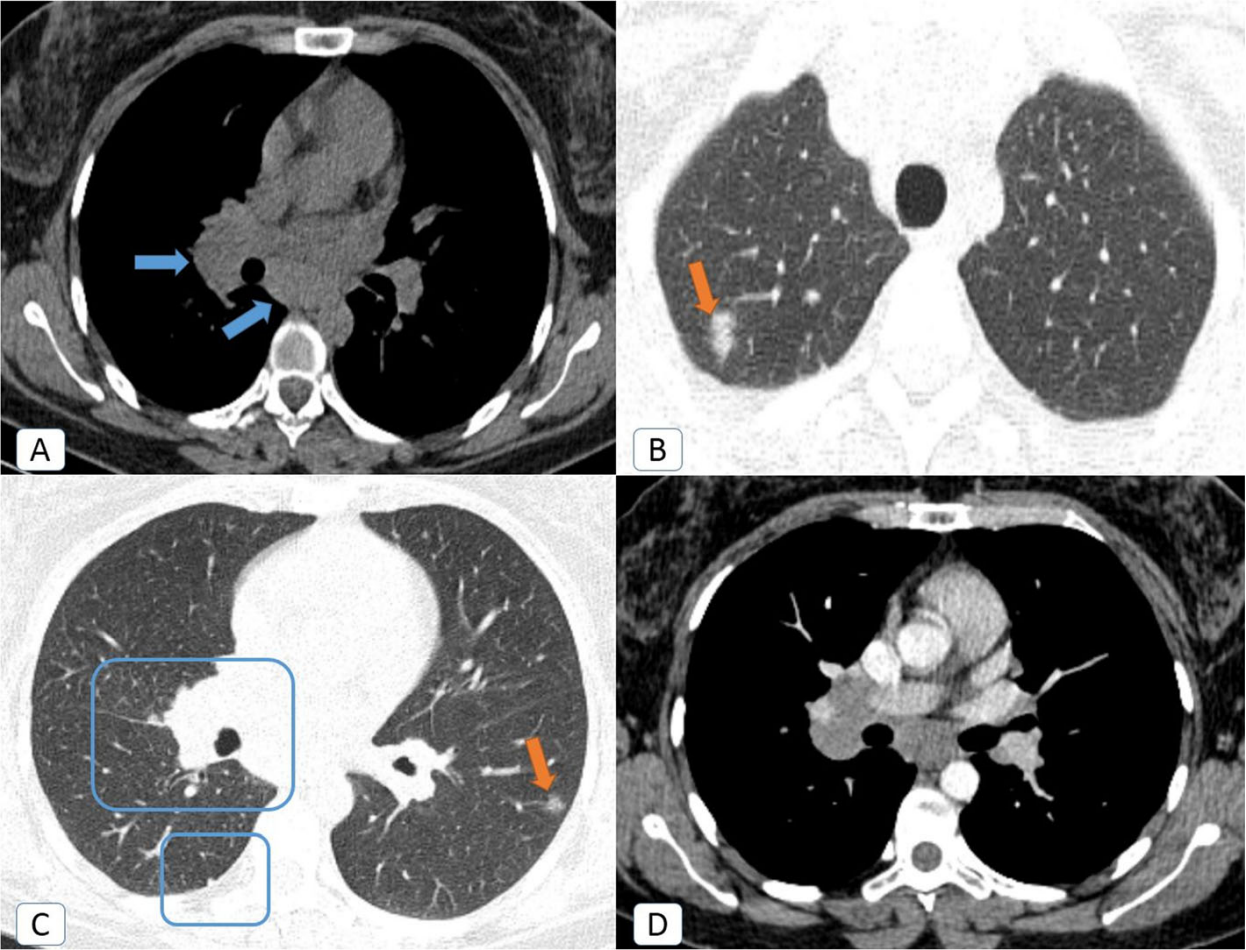
COVID-19 infection on top of incidentally discovered sarcoidosis: a 43-year-old female COVID-19 patient. **A** Initial axial chest CT (mediastinal window) showing enlarged right hilar and sub-carinal lymph nodes (blue arrows), **B** lung window CT cut revealed right apical small ground glass patch (orange arrow), **C** lung window CT at lower level revealed: enlarged right hilar shadow with nearby nodular fissural thickening and lower lobar pleural based nodule (blue squares) as well as left lateral basal sub-pleural small ground glass patch, **D** follow up contrast-enhanced chest CT after one month and resolution of the ground-glass patches showed persistent enlarged mediastinal and right hilar nodes. Nodal biopsy proved sarcoid disease later on

**Fig. 3:**
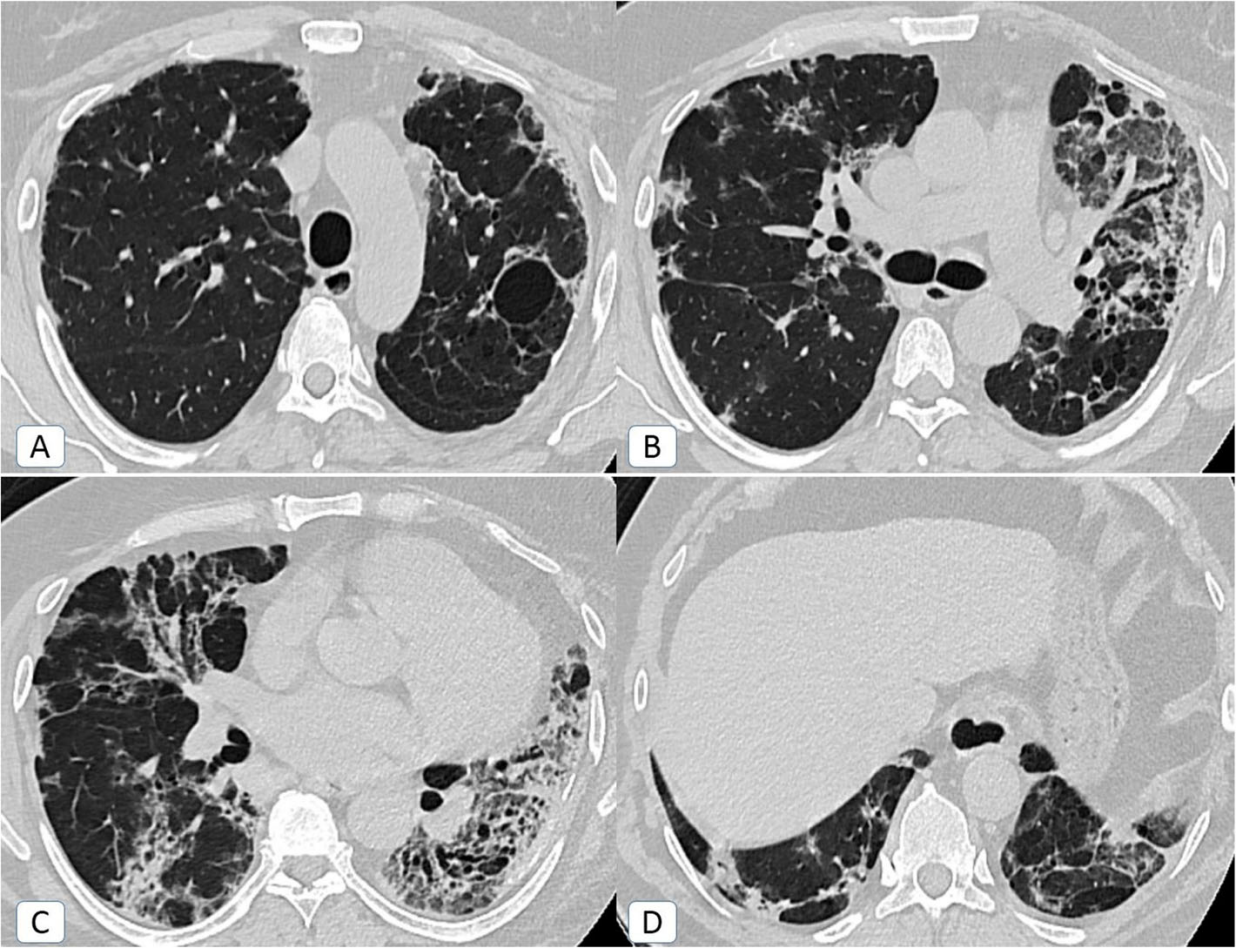
COVID-19 infection on top of old TB and fibro-thorax: a 55-year-old female COVID-19 patient. **A**. Axial chest CT (lung window) showing bilateral apical pleural and sub-pleural fibrotic changes, more accentuated on the left side with cystic changes and centrilobular emphysema. **B**, **C** Axial chest CT (lung window) showing right upper and lower lobar peripheral located ground glass patches of recent COVID-19 infection. This is together with a diminished volume of the left hemithorax which showed ground glass changes, fibrotic changes with traction bronchiectasis and centrilobular emphysema. **D** Axial chest CT (lung window) showing bilateral basal fibro-atelectatic and curvilinear bands

The prevalence rate of preexisting lung comorbidity increased four times throughout the second pandemic wave (significant *P* value = 0.000073). The prevalence rate of COPD and interstitial lung diseases (ILDs) had significantly elevated (*P* value = 0.005 and 0.025, respectively). Despite that the prevalence rate of *sarcoidosis* had doubled, it was statistically insignificant (*P* value = 0.164) (Fig. 2). A single patient with old TB and fibro-thorax was encountered in the second pandemic wave (Fig. 3).

Hospitalization with oxygen support using high-flow nasal cannula up to mechanical ventilation was prescribed for 224/1000 patients (22.4%) during the first pandemic wave and 289/1000 patients (28.9%) during the second wave.

### MSCT findings of the disease (Table 1)

The *ground*-*glass opacities with or without consolidative changes* remain the gold standard CT-findings in all patients throughout the first and second pandemic waves.

During the second pandemic wave, the prevalence rate of the “*crazy*-*paving*” *pattern* and *peri*-*lobular fibrosis with architectural distortion* had significantly increased by 1.3 and 1.4 times respectively (*P* value = 0.002 and 0.02 respectively). Additionally, the prevalence rate of “*air*-*bubble*” *sign* had significantly increased by 1.9 times (*P* value = 0.02). Similarly, the presence of *enlarged mediastinal lymph nodes* (> 1 cm in short-axis diameter) had significantly increased by 1.7 times (*P* value = 0.036). Furthermore, the prevalence rate of *pericardial effusion* had significantly increased by 2.5 times (*P* value = 0.003). The above-mentioned signs were correlated to increased clinical severity. They were encountered in 98% and 93% of hospitalized patients throughout the first and second pandemic waves respectively.

**Fig. 4:**
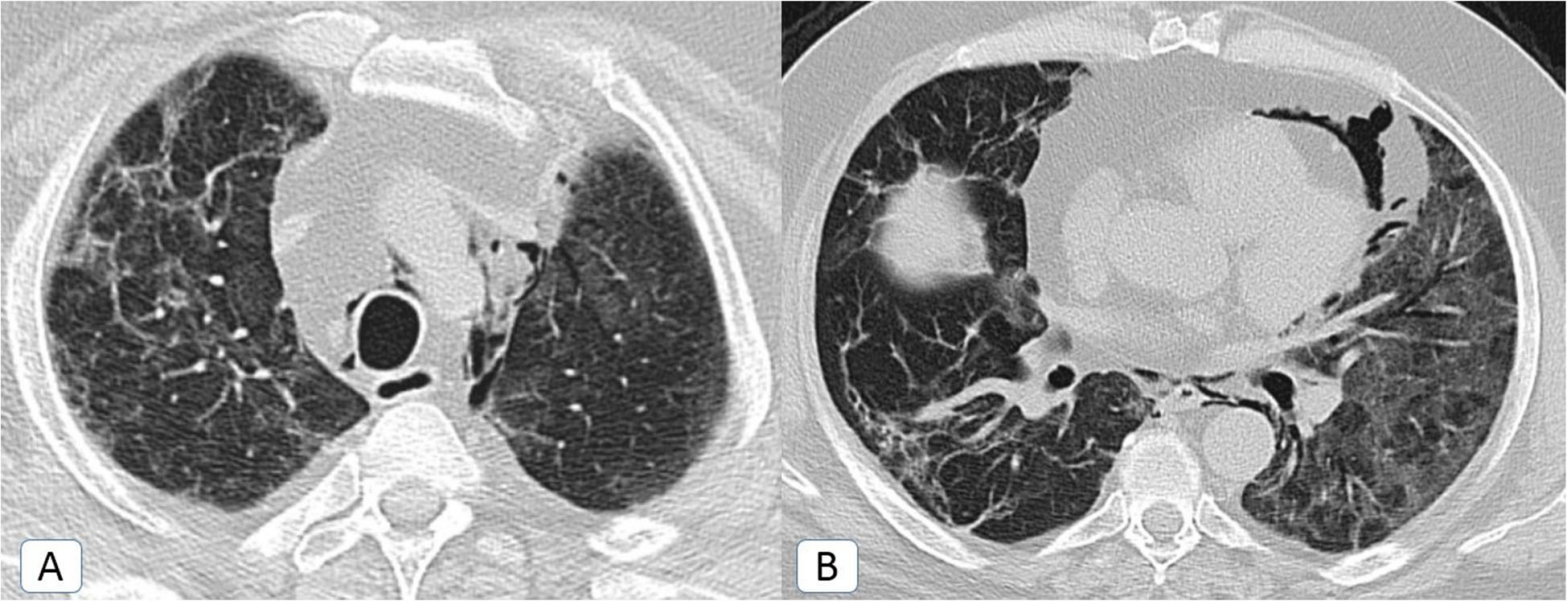
COVID-19 infection complicated by spontaneous pneumo-mediastinum: a 44-year-old male COVID-19 patient. **A**, **B** Axial chest CT (lung window) revealed spontaneous pneumo-mediastinum with bilateral ground-glass patches and fibro-atelectatic bands of COVID-19 infection. Minimal associated pneumo-thorax was also there

*Spontaneous pneumo-mediastinum*, in patients who did not receive oxygen therapy, was encountered only in few patients through the first and second pandemic waves with minimal differences in between (0.2% and 0.7% respectively) (Fig. 4). All patients experienced rapid progressive dyspnea which necessitated hospitalization.

**Fig. 5:**
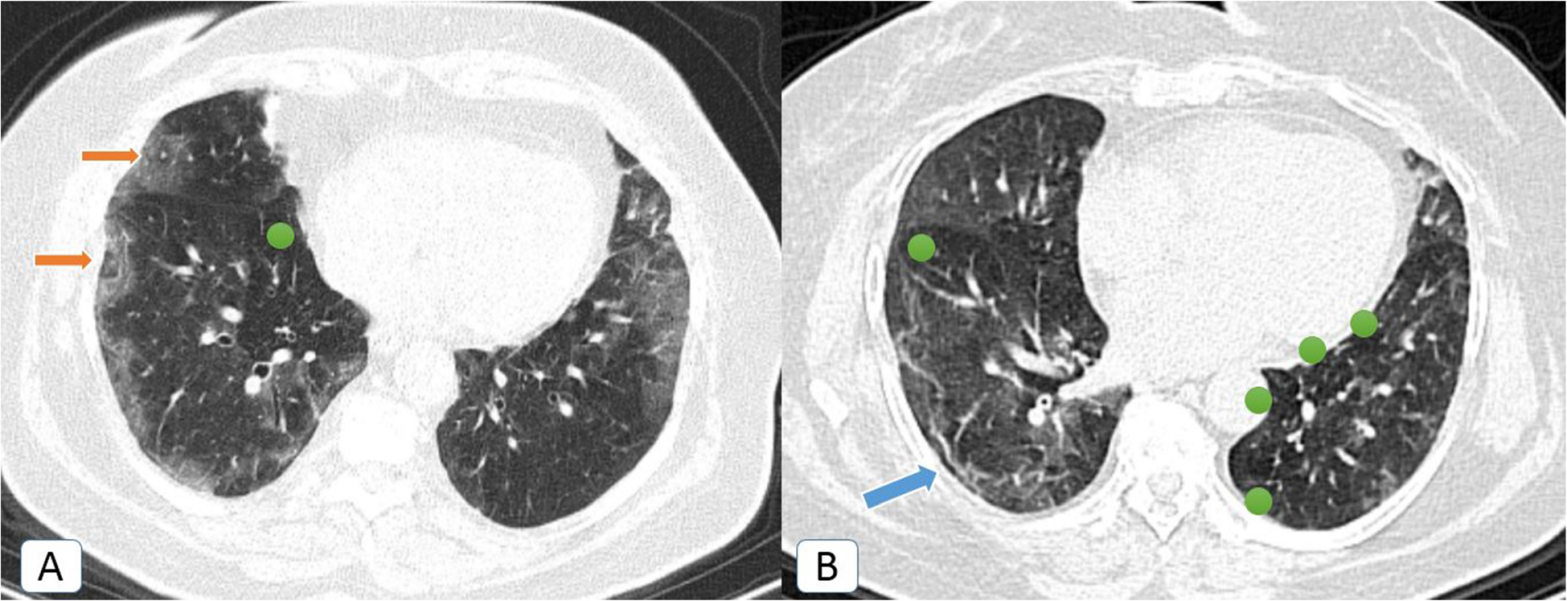
COVID-19 infection atypically presented by head-cheese pattern (ground-glass attenuation alternating with air trapping): a 48-year-old female COVID-19 patient. **A** Initial axial chest CT (lung window) revealed multiple sub-pleural ground glass patches (orange arrows) of COVID-19 infection with a nearby medial basal area of air trapping (green circle) … “Head-cheese pattern.” **B** Follow up axial chest CT after 3 weeks (lung window) revealed healing of the ground-glass patches by curvilinear fibrotic bands (blue arrow) with persistent areas of air trapping

**Fig. 6:**
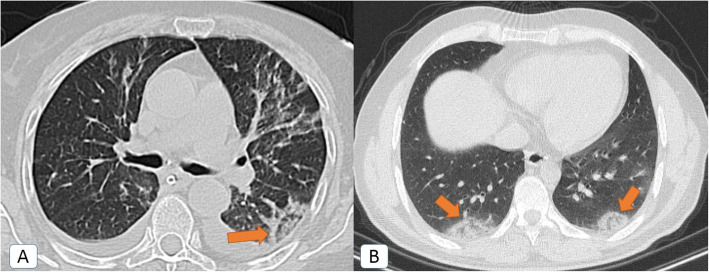
Bulls-eye sign or target sign or double-halo sign: a 36-year-old male COVID-19 patient. **A**, **B** Axial chest CT (lung window) showing bilateral posterior basal sub-pleural patches of central and peripheral consolidations with intervening ground-glass attenuation. This is together with bilateral minimal pleural collection and left lingular mild fibro-atelectatic bands

**Fig. 7:**
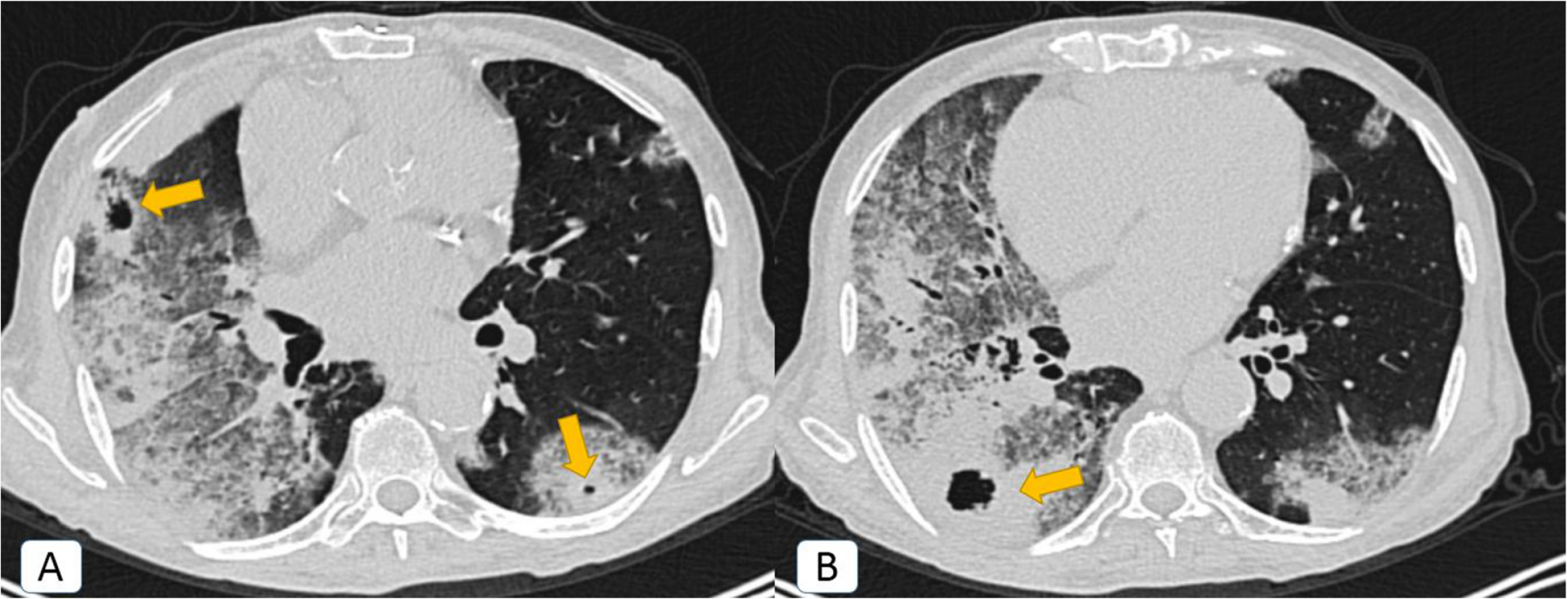
COVID-19 infection atypically presented by cavitation: a 62-year-old male COVID-19 patient. **A**, **B** Axial chest CT (lung window) showing bilateral widespread mixed ground glass and consolidative patches are noted (more pronounced on the right side). Secondary cavitary changes are noted (orange arrows)

Unexpectedly, other *atypical radiological signs* were depicted only during the second pandemic wave, including bronchiectatic changes (2.5%), “head-cheese” pattern (0.8%) (Fig. 5), “bulls-eye” sign (0.2%) (Figs. 5 and 6), and cavitation (0.5%) (Fig. 7).

**Fig. 8:**
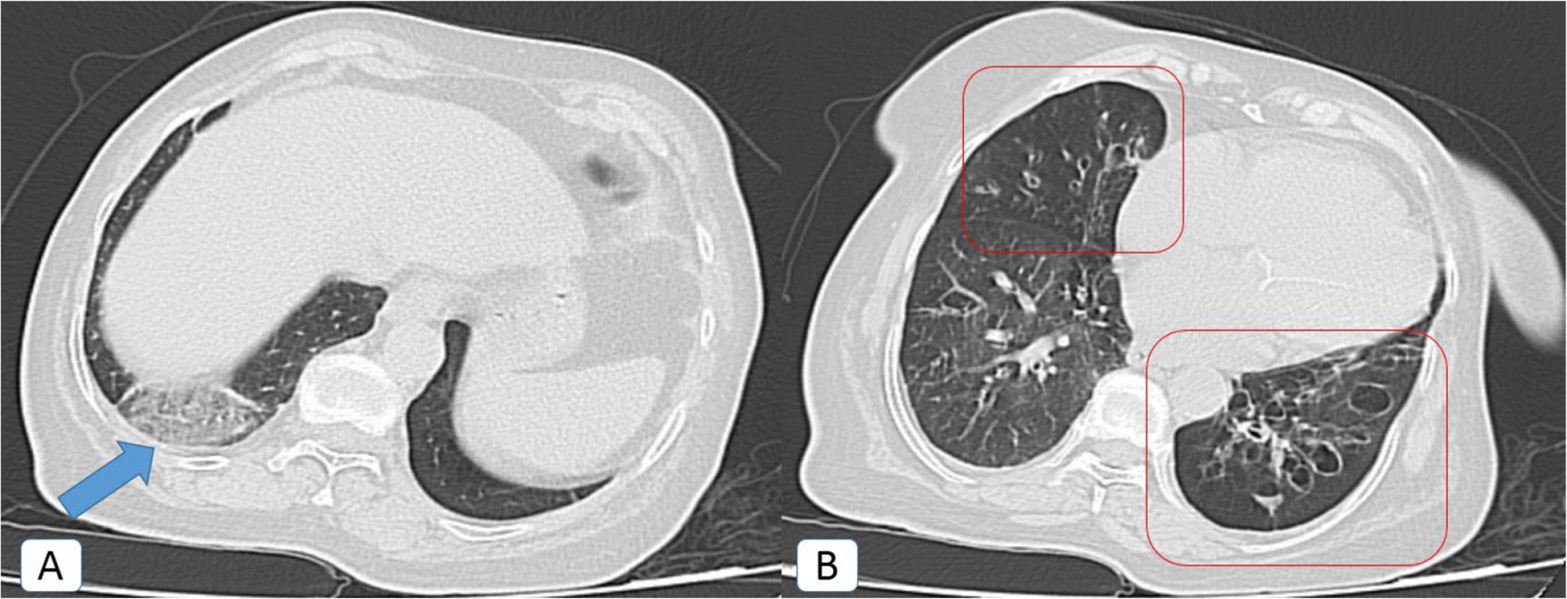
COVID-19 infection atypically presented by cystic bronchiectasis: a 58-year-old female COVID-19 patient without a history of previous chest disease. **A** Axial chest CT (lung window) showing right basal sub-pleural ground glass patch (blue arrow). **B** Axial chest CT (lung window) showing right middle lobar traction bronchiectatic changes and left lower lobar cystic bronchiectatic changes with air-fluid levelling and air trapping (red squares)

The bronchiectatic changes were cylindrical in type sequel to early fibro-atelectatic changes (traction bronchiectasis) in 22/25 patients. Meanwhile, cystic bronchiectasis was encountered in three patients (Fig. 8).

**Fig. 9:**
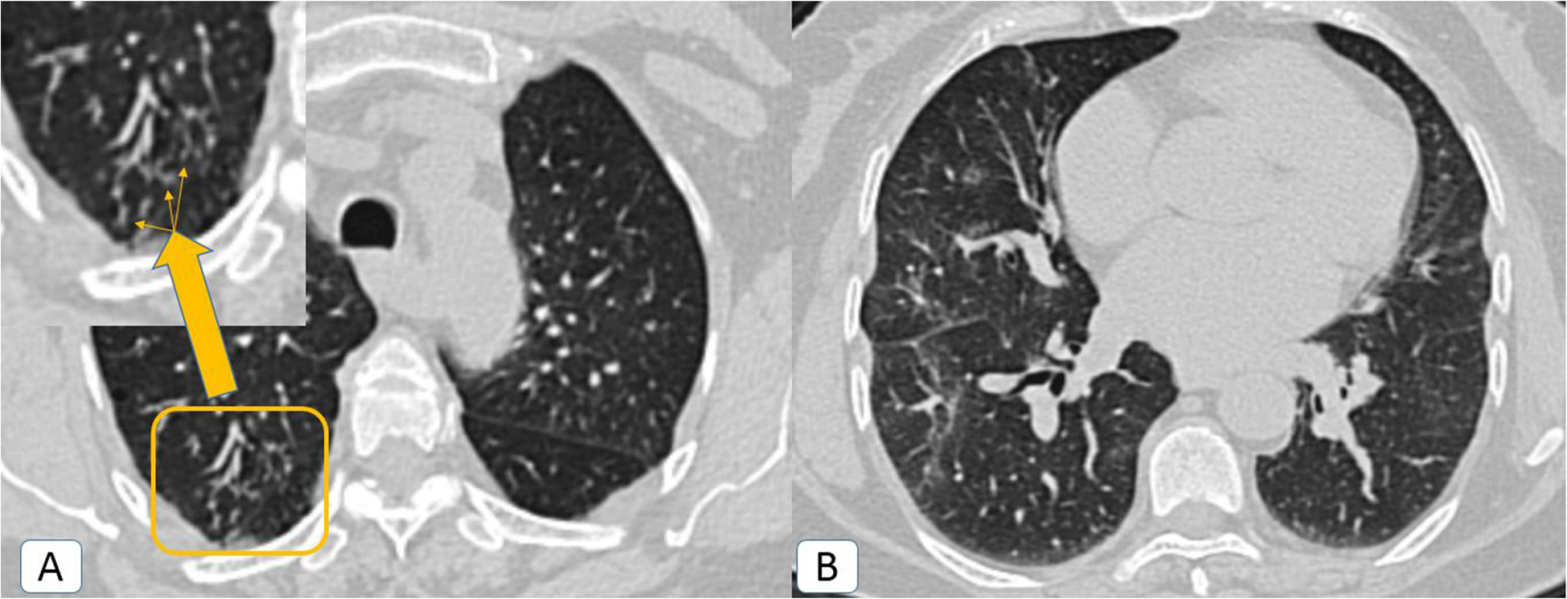
COVID-19 infection atypically presented by tree in bud nodules: a 71-year-old female COVID-19 patient**. A** Axial chest CT (lung window) showing small tree in bud branching peripheral sub-pleural micro-nodules in the posterior segment of the right upper lobe (yellow square and yellow arrow pointing to zoomed image). **B** Axial chest CT (lung window) showing bilateral ground-glass patches as well

Tree in bud nodules that represent bronchiolitis were similarly encountered once throughout the first and second pandemic waves (Fig. 9).

### Secondary infection (Table 1)

**Fig. 10:**
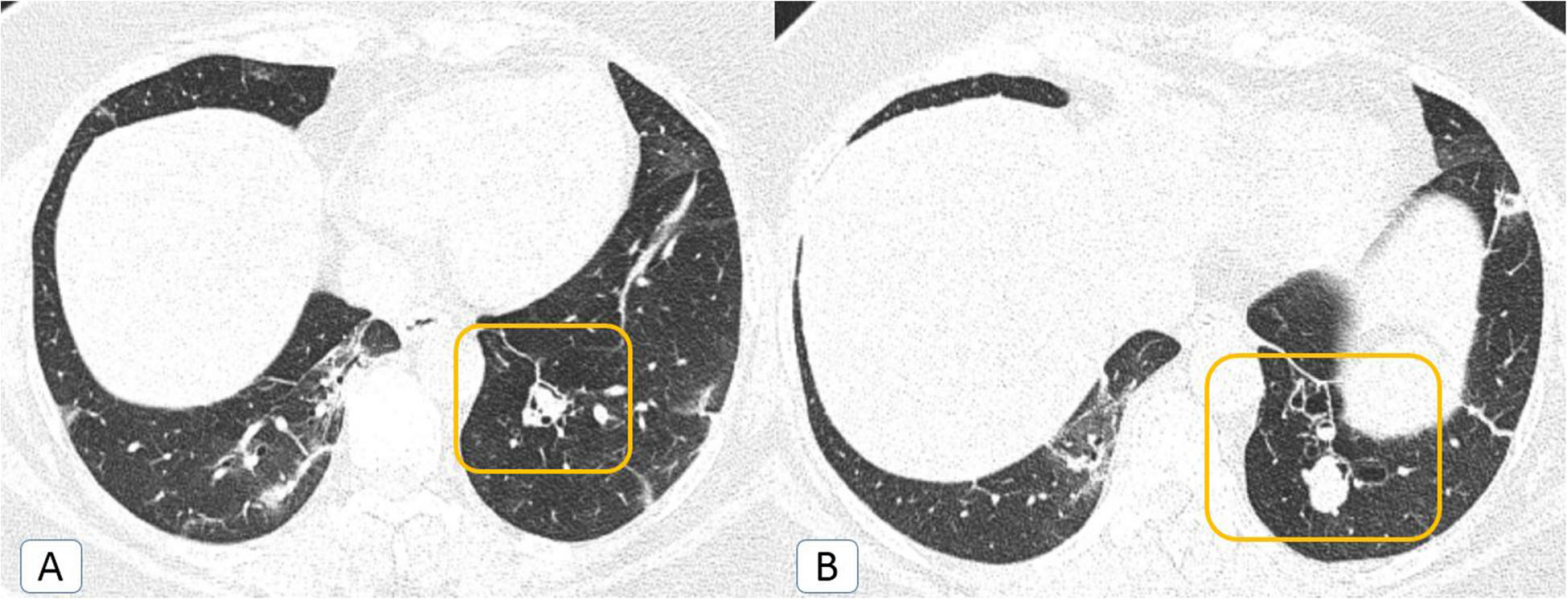
COVID-19 infection complicated by secondary fungal infection: a 49-year-old male COVID-19 patient without a history of previous chest disease. **A** Axial chest CT (lung window) showing bilateral basal sub-pleural ground glass patches and fibro-atelectatic bands of COVID-19 infection together with left basal small cavitary lung lesion with eccentric rounded soft tissue density (fungus ball or mycetoma) with eccentric air (air-crescent sign). **B** Axial chest CT (lung window) additionally left basal cystic bronchiectatic changes with mucous plugging and air-fluid leveling

Four patients (0.4%) with secondary fungal infection were only documented during the second pandemic wave (significant *P* value = 0.045). Two patients of them had typical mycetoma balls (non-invasive pattern), while one patient had positive CT signs of semi-invasive aspergillosis including mucous plugging, air-fluid leveling, and air-trapping. One patient had mixed non-invasive and semi-invasive CT findings (Fig. 10). Sputum culture confirmed the diagnosis and anti-fungal therapy was prescribed.

### Post-infectious sequel or complications (after 1 month of infection) (Table 1)

Follow up CT examinations were only performed for 49 and 122 patients through the first and second pandemic waves respectively.

*Persistent ground*-*glass opacities* were encountered in 7/49 (14.3%) and 41/122 (33.6%) patients respectively.

**Fig. 11:**
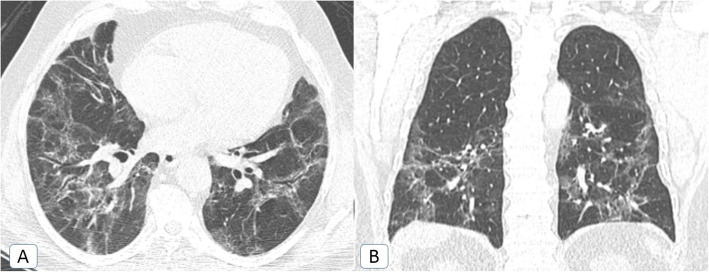
Post-COVID fibrosis: a 57-year-old male COVID-19 patient. Chest CT was done 3 months after the COVID-19 infection. **A** Axial MPR (lung window) and **B** coronal MPR (lung window), showing bilateral upper and lower lobar persistent ground-glass patches and irregular fibrotic changes with parenchymal distortion and mild traction bronchiectatic changes

The prevalence rate of *post*-*COVID fibrosis* had doubled through the second pandemic wave but not in a significant way (*P* value = 0.234) (Fig. 11).

Persistent dyspnea was documented in 71% and 79% of patients with persistent ground-glass opacities and post-COVID fibrosis. They had received long term steroid therapy and scheduled for long term clinical and radiological follow up.

### COVID-19 re-infection

**Fig. 12:**
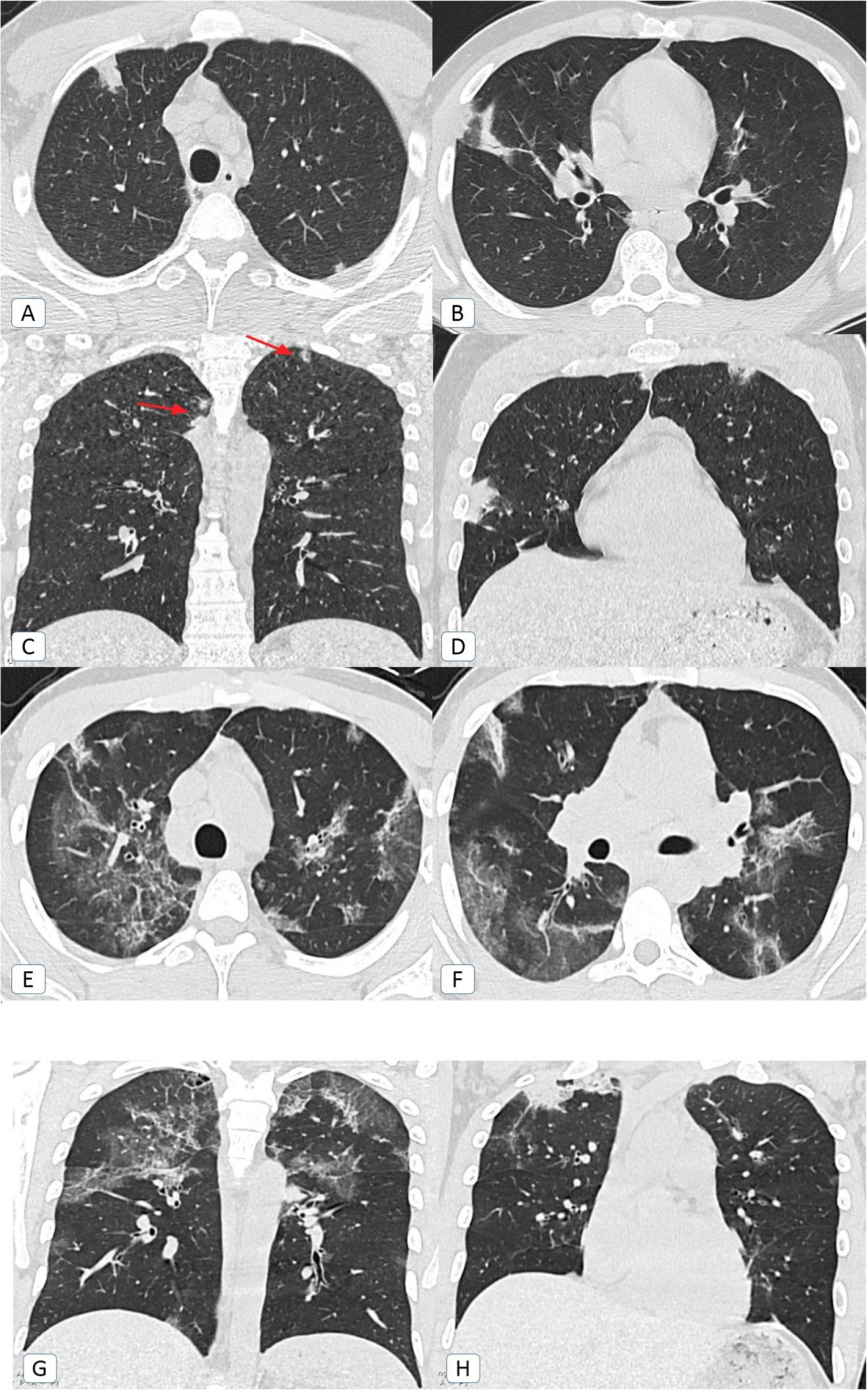
COVID-19 reinfection: A 35-year-old male COVID-19 patient. **A**, **B** Axial, and **C**, **D** coronal chest CT (lung window) during the mild first infective episode at the beginning of October 2020, showing small bilateral upper lobar mixed ground glass and consolidative patches. **E**, **F** Axial and **G**, **H** and coronal chest CT (lung window) during the more severe reinfection at the end of March 2021, showing widespread bilateral upper and lower lobar mixed ground glass and consolidative patches with fibro-atelectatic changes. Also noted right apical mild cystic changes as a sequel to the previous infection

A single patient with COVID-19 re-infection was encountered during the second pandemic wave. He had his first infection at the beginning of October 2020 and re-infection around 6 months later at the end of March 2020 (Fig. 12).

## Discussion

According to the Egyptian Ministry of health, the second pandemic wave of COVID-19 had stricken Egypt during the period from November 2020 till January 2021 (https://www.care.gov.eg). This period coincided with the winter season and followed the gradual re-opening of schools and universities. This fact could explain the striking elevation in the infection rate between the young Egyptian people in the second decade of life as well as the surge of infection between the children. This is similar to the findings in the study by Park JE et al. [18], in which low temperature and low humidity of the winter season were responsible for the more severe course of viral infections.

At first, the authors thought that the virus predilects non-pathological lung parenchyma because of the very low incidence of preexisting lung comorbidity. But a significant rise of this incidence throughout the second pandemic wave was striking. The pulmonologists in this study tried to explain this notice. They hypothesized the following scenario. Meanwhile, those patients were considered as extremely vulnerable patients at high risk for COVID-19 complications, hence their physicians gave them strict warnings about the importance of COVID-19 precautions and this may decrease its incidence. Additionally, they minimized or delayed their chest CT needs to very limited situations (for fear of inadequate infection control measures). Even if their patients had suspicion about COVID-19, they prefer to depend on laboratory diagnosis with PCR instead of chest CT because radiological findings between the original disease and COVID-19 may overlap. They almost limit the role of CT to those patients with suspected false negative PCR results or prior to hospitalization if needed. This can explain the very low incidence the first pandemic wave, but off course by time, the need for chest CT examinations increased and the confidence about infection control measures raised.

Emphysema and ILDs were encountered during the second pandemic wave. Hussain MR et al. [19] was a case report study that similarly discussed the exacerbation of ILDs in a COVID-19 patient. Additionally, the incidental diagnosis of sarcoid disease was significantly reported in this study, which followed the wide use of chest CT examinations for either suspected or diseased COVID-19 patients. A single case of old TB was only reported in the second pandemic wave. Miller A et al. [20] hypothesized a theory about the immunity of people who received compulsory BCG vaccine against COVID-19 infection, but this study had not peer-reviewed and this theory had not been proved till writing this manuscript.

Coinciding with the universal criteria of COVID-19 radiological diagnosis [12, 13], the typical CT signs of COVID-19 infection were encountered in this study, including ground-glass opacities (GGOs) with or without consolidative changes. This is consistent with the findings of other previous CT studies including Omar S et al. [21], Ali TF et al. [22], Emara DM et al. [23], Sabri YY et al, [24], and Mohamed IA et al. [25].

In this study, the clinical severity through the second pandemic wave had increased by around 6.5%. This coincided with the higher rates of pulmonary and extra-pulmonary comorbidities or complications in addition to the increased prevalence of five CT findings of severity, including the “crazy-paving” pattern, “air-bubble” sign, significant mediastinal nodal enlargement, pericardial effusion, and spontaneous pneumo-mediastinum. Previous studies described the “crazy-paving” pattern in 100% of their severe patients, including Ali TF et al. [22], Ghweil AA et al. [26], Metwally M.I et al. [27], and Osman AM et al. [28]. Additionally, Li Y et al. [29] correlated the presence of pericardial effusion to the high clinical severity. Wang J et al. [30] also stated that 100% of patients with spontaneous pneumo-mediastinum suffered from severe respiratory distress and needed hospitalization. According to Lei P et al. [31], the spontaneous pneumo-mediastinum was encountered at the time of the resolution of the ground-glass opacities or consolidations and their replacement by peri-bronchiolar fibrosis or abscess formation with secondary interstitial emphysema.

In this study, some atypical CT findings were only encountered during the second pandemic wave. They included the bronchiectatic changes, the “head-cheese” pattern, the “bulls-eye” sign, and the cavitation. Therefore, the authors believe that radiologists everywhere should not exclude COVID-19 infection entirely if they found these CT findings.

The traction bronchiectatic changes, in this study, coincided with the early peri-lobular fibrosis and parenchymal distortion with high clinical severity. This was similar to the findings in the studies by Sabri YY et al. [2] and Zhao W et al. [32].

This study agreed with the recent literature in 2021 that first described uncommon cavitary changes in COVID-19 patients, including Ammar A et al. [33], Zoumot Z et al. [34], Selvaraj V et al. [35], Özgül HA et al. [36], and Afrazi A et al. [37].

In this study, the second pandemic wave witnessed a doubling rise regarding the incidence of post-COVID fibrosis. Despite that, this rise was statistically insignificant. The post-COVID fibrosis throughout the first and second pandemic waves was correlated to the persistent dyspnea after the announcement of patient recovery. However and unexpectedly, 21–29% of patients with radiologically persistent lung fibrosis were asymptomatic. Spagnolo P et al. [38] and Tale S et al. [39] alarmed also to the bad prognosis of post-COVID fibrosis. Udwadia ZF et al. [40] dramatically described it as the tsunami that follows the earthquake.

The super-added fungal infection at the second pandemic wave included either non-invasive or semi-invasive patterns. It is mostly explained by the mild decrease in lung immunity. It could be an additional explanation for the increased clinical severity.

This study adds to the literature the radiological differences between the first and second pandemic waves in one of the largest countries in the Middle East, Egypt, highlighting important unexpected findings.

This study was limited by the short time interval after subside of the second pandemic wave in Egypt; hence, further future researches about its long term effect are recommended.

## Conclusion

After one year from the announcement of COVID-19 as a pandemic, the radiological presentation of COVID-19 patients showed some significant differences between its first and second waves.

## Data Availability

The datasets used and/or analyzed during the current study are available from the corresponding author on reasonable request.

## Abbreviations

COVID 19: Coronavirus disease 2019
MSCT: Multi-slice computed tomography
MPR: Multi-planar reconstruction
MIP: Maximum intensity projection
MinIP: Minimum intensity projection
